# Reproductive health for refugees by refugees in Guinea II: sexually transmitted infections

**DOI:** 10.1186/1752-1505-2-14

**Published:** 2008-10-23

**Authors:** Mark I Chen, Anna von Roenne, Yaya Souare, Franz von Roenne, Akaco Ekirapa, Natasha Howard, Matthias Borchert

**Affiliations:** 1 London School of Hygiene and Tropical Medicine, London, UK; 2 Gesellschaft für Technische Zusammenarbeit, Eschborn, Germany; 3 Reproductive Health Group, Guéckédou, Guinea

## Abstract

**Background:**

Providing reproductive and sexual health services is an important and challenging aspect of caring for displaced populations, and preventive and curative sexual health services may play a role in reducing HIV transmission in complex emergencies. From 1995, the non-governmental "Reproductive Health Group" (RHG) worked amongst refugees displaced by conflicts in Sierra Leone and Liberia (1989–2004). RHG recruited refugee nurses and midwives to provide reproductive and sexual health services for refugees in the Forest Region of Guinea, and trained refugee women as lay health workers. A cross-sectional survey was conducted in 1999 to assess sexual health needs, knowledge and practices among refugees, and the potential impact of RHG's work.

**Methods:**

Trained interviewers administered a questionnaire on self-reported STI symptoms, and sexual health knowledge, attitudes and practices to 445 men and 444 women selected through multistage stratified cluster sampling. Chi-squared tests were used where appropriate. Multivariable logistic regression with robust standard errors (to adjust for the cluster sampling design) was used to assess if factors such as source of information about sexually transmitted infections (STIs) was associated with better knowledge.

**Results:**

30% of women and 24% of men reported at least one episode of genital discharge and/or genital ulceration within the past 12 months. Only 25% correctly named all key symptoms of STIs in both sexes. Inappropriate beliefs (e.g. that swallowing tablets before sex, avoiding public toilets, and/or washing their genitals after sex protected against STIs) were prevalent. Respondents citing RHG facilitators as their information source were more likely to respond correctly about STIs; RHG facilitators were more frequently cited than non-healthcare information sources in men who correctly named the key STI symptoms (odds ratio (OR) = 5.2, 95% confidence interval (CI) 1.9–13.9), and in men and women who correctly identified effective STI protection methods (OR = 2.9, 95% CI 1.5–5.8 and OR = 4.6, 95% CI 1.6–13.2 respectively).

**Conclusion:**

Our study revealed a high prevalence of STI symptoms, and gaps in sexual health knowledge in this displaced population. Learning about STIs from RHG health facilitators was associated with better knowledge. RHG's model could be considered in other complex emergency settings.

## Background

Displaced populations continue to need reproductive and sexual health services during armed conflicts, which can last for decades [1]. The provision of reproductive and sexual healthcare in populations affected by complex emergencies poses a unique challenge. Behavioural changes arising from large population movements, social disruption and the poverty and violence experienced by displaced persons, may increase incidence of sexually transmitted infections (STIs) and HIV [2,3]. Men may have opportunistic sex or visit sex workers [4]. Women may be raped, coerced to trade sex, or enter relationships to secure basic survival [3,5]. However, there is evidence that the impact of conflict on the sexual transmission of HIV is context specific [6-9], depending on factors such as the prevalence of STIs and HIV in the populations involved and the adequacy of relevant refugee health services [10]. Health programmes must thus adapt their approaches to provide prevention and treatment services specific to the setting [11].

There has been a lack of published epidemiological research dealing with the implementation of sexual and reproductive health interventions for displaced populations [2]. Here we report about a knowledge, attitude and practice survey among Sierra Leonean and Liberian refugees of reproductive age, residing in camps in Guinea, where the "Reproductive Health Group" (RHG) had provided reproductive health services to refugees for several years. RHG, a local non-governmental organisation, is of special interest because they recruited nurses and midwives from the refugee community itself, and seconded them to Guinean health facilities, while trained refugee lay women provided contraceptives and health education, and drama groups attempted to specifically reach males and adolescents. Details on RHG's activities and the setting where they were active are described in the companion paper [12] and elsewhere [13].

The survey was conducted in 1999 and had the following objectives:

• To assess sexual health needs, knowledge and practices among refugees, e.g. prevalence of reported STI symptoms, knowledge about symptoms and prevention of STIs, treatment seeking and protective behaviour adopted by those experiencing STI symptoms

• To assess the potential impact of RHG's work, in terms of increased STI knowledge and more appropriate STI-related behaviour in RHG clients

Survey results on family planning aspects are reported in the companion paper [12].

## Methods

Details on study population, sampling strategy, survey methodology, data entry and analysis are provided in the companion paper [12]. In brief, a cross-sectional survey was conducted in a representative multistage sample of 889 reproductive-age men and women refugees from 48 camps served by RHG. In addition to socio-demographic information, survey sections of relevance here include questions on self-reported STI symptoms, and STI-related knowledge and behaviour. Respondents who had some knowledge of STIs were asked to name, unprompted, STI symptoms in men and women, and to assess a list of measures for protecting themselves from STIs – this list included two accepted and effective methods, and three inappropriate but prevalent methods. They were also asked about their main information source (e.g. health workers, RHG facilitators, dramas, friends, family, or media). In addition, respondents who reported genital discharge and/or ulceration within the past 12 months were asked about their treatment-seeking and partner notification/protection measures.

Study outcomes were:

• correctly identifying genital discharge AND genital ulcers in men and women as STI symptoms (key symptoms validated in African settings for syndromic STI management) [14]

• correctly identifying two accepted methods as effective for protecting against STIs (staying with one faithful partner, or using condoms during sexual intercourse)

• NOT agreeing that three inappropriate methods for protecting against STIs were effective (swallowing a tablet before sexual intercourse, avoiding public toilets, or – for women – washing their genitals after sexual intercourse)

• adopting an appropriate combination of behaviours when having STI symptoms, namely notifying their partner/s about their STI symptoms (which would facilitate care-seeking by the partner/s), AND protecting the partner/s from potential acquisition of an STI (either by abstaining from sex, or by using a condom)

Prevalence of these outcomes was compared between groups citing different sources of STI information and associations tested between outcome variables and socio-demographic and behavioural covariates. Chi-squared tests were used where appropriate. Covariates significant at p < 0.10 were entered into a multivariable logistic regression model. Robust standard errors were used to account for cluster sampling.

The study received ethical clearance from the Ministry of Public Health in Guinea and the London School of Hygiene & Tropical Medicine in the UK.

## Results

The response rate exceeded 95%, and the final sample used was 889 (445 men and 444 women). Results on demographics are reported in the companion paper [12].

### STI knowledge

Most respondents (90% of men, 92% of women) had some knowledge of STIs (Table 1). 43% of men and 58% of women (Chi2-test, p < 0.001) cited RHG facilitators as their main source of STI information, while 18% of respondents cited healthcare workers and 7% RHG drama groups. Men indicated non-healthcare sources more frequently than women (22% vs. 10%, p < 0.001; 18% vs. 9% named friends and family as their main source of STI information, 4% vs. < 1% radio programmes or school). Self-reported STI symptoms were common, with 30% of women and 24% of men (p = 0.02) reporting at least one episode of genital discharge and/or ulceration within the past 12 months.

**Table 1:** Source of STI knowledge and prevalence of self-reported STI symptoms

	Men, N = 445	Women, N = 444	
			
Characteristic	% (n)	% (n)	p-value (Chi^2 ^test)
Ever heard of STIs	89.7% (399)	92.3% (410)	0.163
			
Most important source of information about STIs			<0.001
*Healthcare and RHG sources*			
- health workers	17.1% (76)	18.2% (81)	
- RHG drama groups	7.2% (32)	6.3% (28)	
- RHG health facilitators	43.4% (193)	57.7% (256)	
*Non-healthcare sources*			
- friends and family	18.2% (81)	9.5% (42)	
- radio	2.7% (12)	0.2% (1)	
- school	0.9% (4)	0.2% (1)	
- others	0.2% (1)	0.2% (1)	
			
Had STI symptoms in the past 12 months			
- had genital discharge	21.8% (97)	26.6% (118)	0.096
- had a genital ulcer	8.8% (39)	12.4% (55)	0.079
- had either genital discharge or a genital ulcer	23.6% (105)	30.4% (135)	0.022
- had both genital discharge and a genital ulcer	7% (31)	8.6% (38)	0.375

**Figure 1:**
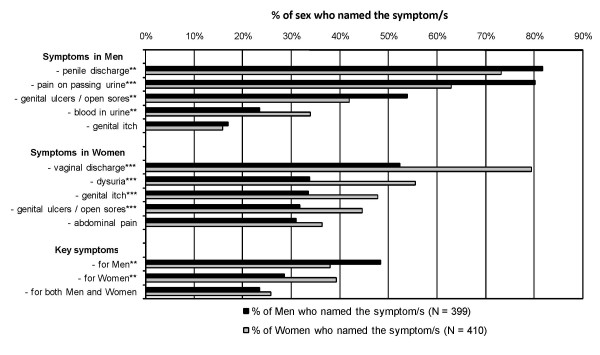
**Knowledge of STI symptoms in men and women, by sex of respondent**. The 399 men and 410 women who had ever heard of STIs were asked to name, without prompting, STI symptoms in men and women. Results for the five STI symptoms most frequently named are presented; we also present the proportion that name a combination of key STI symptoms in men, in women, and in both men and women. Key symptoms were defined as penile discharge and genital ulcers/open sores in men, and vaginal discharge and genital ulcers/open sores in women. Items where the proportions differ significantly between genders are annotated (*** p < 0.001, ** p < 0.01, * p < 0.05).

Figure 1 presents the results on knowledge of STI symptoms, showing the five STI symptoms most frequently named by the 399 men and 410 women who had some knowledge of STIs. Both sexes were more familiar with STI symptoms in their own rather than the opposite sex. While around 75% of respondents recognised penile discharge as an STI symptom, only 52% of men recognised vaginal discharge as a possible STI symptom for women. Only 32% of men and 42% of women suggested genital ulcers as STI symptoms in the opposite sex. Failure to recognise genital ulcers as an STI symptom in the opposite sex meant that only a minority of those who had some knowledge of STIs could correctly name the two key STI symptoms of genital discharge and genital ulceration for both sexes (24% of men and 26% of women, respectively).

Table 2 (see additional file 1) presents STI prevention findings in individuals with some knowledge of STIs. Most respondents recognised that staying with one faithful partner and using condoms were effective, and 86% of men and 89% of women agreed with both methods. However, almost two thirds also judged one or more inappropriate STI prevention beliefs (e.g. swallowing tablets before sex, avoiding public toilets, and/or washing their genitals after sex) to be effective; only 38% of men and 41% of women rejected all inappropriate suggestions.

### STI treatment-seeking behaviour

**Figure 2:**
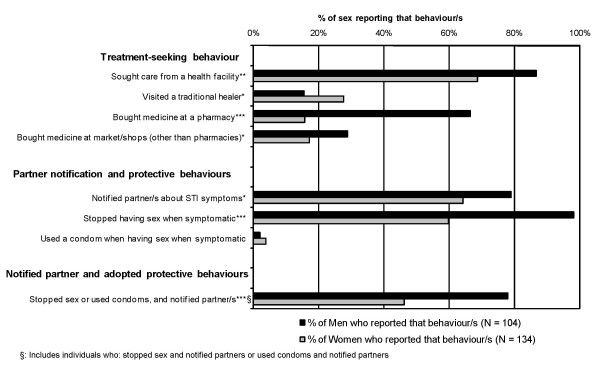
**Treatment-seeking and partner notification/protection among symptomatic individuals, by sex of respondent**. The 104 men and 134 women who reported genital discharge and/or ulceration in the past 12 months were asked if they adopted any of these behaviours when having STI symptoms; note that each respondent could report more than one behaviour. We also present the proportion that adopted a combination of appropriate behaviours – either stopping sexual intercourse or using a condom, plus notifying their partner regarding their symptoms. Items where the proportions differ significantly between genders are annotated (*** p < 0.001, ** p < 0.01, * p < 0.05).

Figure 2 presents the behaviours reported by individuals when experiencing genital discharge and/or ulceration. Men were more likely than women to seek care from health facilities or to purchase medicines. Women were more likely than men to visit a traditional healer. Men were also more likely than women to notify their partners or avoid sex when symptomatic. Condom use, to protect partners when symptomatic, was infrequently reported by either sex. Approximately 78% of men adopted the appropriate combination of behaviours when perceiving STI symptoms, i.e. they reportedly informed their partner/s, and protected them from potential acquisition of the STI, mostly by stopping sex, less frequently by using a condom. This percentage was much lower in women (46%).

### Outcome indicators by source of STI information

Table 3 (see additional file 2) presents the prevalence of selected outcome indicators for men and women, grouped by STI information source. Among respondents who had some knowledge of STIs, correct knowledge about STI symptoms and prevention was significantly less frequent in those citing non-healthcare sources, while those citing RHG facilitators as their main information source were more likely to respond correctly. Among those with STI symptoms, respondents citing RHG facilitators as their main source of knowledge were more likely to adopt appropriate behaviours than those citing non-healthcare sources, although this difference was not statistically significant. Several outcomes remained significantly associated with STI information source after adjusting for potential confounding in multivariable analyses. Both men and women who cited RHG facilitators were more likely to identify effective STI protection methods (adjusted odds ratio (OR) = 2.9, 95% confidence interval (CI) 1.5–5.8 in men, and OR = 4.6, 95% CI 1.6–13.2 in women) after adjusting for other factors significantly associated with this outcome (educational level and marital status in men, and educational level and age at first sexual intercourse in women). Respondents of both sexes who agreed with the two accepted methods of STI prevention were also marginally (but not significantly) more likely to identify key STI symptoms. Men citing RHG facilitators were significantly more likely to name the key STI symptoms (OR = 5.2, 95% CI 1.9–13.9) when compared with those citing non-healthcare information sources. Women citing RHG facilitators were likewise more likely to name the key STI symptoms, but this association was not statistically significant (OR = 2.0, 95% CI 0.9–4.6, p = 0.106), since levels of knowledge in women citing non-healthcare information sources was higher than for the men (17.8% in women vs 8.2% in women, see Table 3). None of the sociodemographic or behavioural factors (age, religion, educational level, time in refugee camp, marital status and age at first sexual intercourse) were found to be associated with better knowledge about STI symptoms in either men or women.

## Discussion

This survey, conducted under difficult conditions in a refugee population, highlights the necessity of good sexual health services for refugees. The majority of those displaced were sexually experienced; the prevalence of self-reported STI symptoms was high, in line with the high prevalence of lab-confirmed STIs in Rwandan refugees [15]. Similarly high estimates have been reported in reproductive-age women in non-emergency African settings [16].

Most respondents had heard of STIs, but many had only a superficial understanding. Less than 30% identified the two key symptoms in both sexes. While genital discharge was generally known, genital ulcers (more important in facilitating HIV transmission [17]) were rarely named. Although most respondents correctly identified effective STI protection methods, the high prevalence of inappropriate beliefs echoed findings in other studies [4]. Some of these beliefs, such as avoiding public toilets, are clearly ineffective. Other beliefs, like swallowing a tablet before sex, could be effective in some circumstances (eg. antibiotics taken as prophylaxis against bacterial STIs [18]), but ineffective in others, and are therefore inappropriate; such a practice is also inappropriate because the misuse of drugs can cause adverse drug reactions and foster the emergence of resistance [19]. Moreover, believing in inappropriate methods may distract from accepted preventive measures, particularly if the inappropriate methods for protecting against STIs require less effort than the more effective ones (e.g. swallowing a tablet before sex or washing their genitals after sex versus condom use or faithfulness). Thus, community health education must not only inform about effective protection, but also dispel common but inappropriate beliefs about STI transmission.

Gender disparities were noted in treatment-seeking and partner notification. Underlying reasons why men more often purchased medications or accessed health facilities for STI symptoms while women favoured traditional healers may relate to the lack of financial resources available to women in a largely traditional society, but we did not explore this further in our study. Women were half as likely to notify their partners or adopt protective behaviours (mainly sexual abstinence) when symptomatic, suggesting they may have felt disempowered within sexual partnerships. Similar gender disparities reported in non-conflict African settings [20] further support the need for sexual health services and education to address the concerns of both men and women refugees. Gender disparities were similarly detected in the section of our survey dealing with knowledge and attitudes towards family planning [12].

Findings suggest that RHG's health education activities were effective. The survey, undertaken four years after RHG began, showed that RHG had gained sufficient credibility in this displaced population to be cited by most respondents as their main STI information source. Moreover, those who cited RHG facilitators were more likely to know key STI symptoms and effective STI prevention methods, and were less likely to maintain/cite inappropriate STI-related beliefs.

The study was subject to several limitations. First, no laboratory confirmation was possible for reported STI symptoms. However, syndromic STI identification has reasonable positive predictive value for STIs in high prevalence non-conflict African settings [14], and perceived STI is more relevant for health care seeking than actual STI. Second, reverse causality must be considered in cross-sectional studies. One could argue that individuals did not become more knowledgeable through using RHG's services, but used RHG's services because they were more knowledgeable from the outset. Last, care needs to be taken when applying the findings of this study and the wider work concerning RHG's model for reproductive health to other conflict settings, as the services required in any complex emergency are determined by interactions between sexual health risks and needs among refugees, context of the conflict, and characteristics of the host country [10,11].

## Conclusion

Study findings reveal important gaps in sexual health knowledge, high burden of STI symptoms and insufficient access to STI services. The findings suggest the necessity and effectiveness of RHG's intervention model in this refugee population, and similar strategies could work in comparable contexts. In line with *IAWG Field Manual on Refugee Reproductive Health *guidelines, for refugees to participate in designing, maintaining and evaluating their own reproductive health services [21], the authors believe nurses, midwives and laywomen from the refugee community can provide essential reproductive health education and services to their fellow refugees. If properly supported, such programmes are more likely to be accepted and understood by the refugee community. This study contributes to the literature indicating such programmes are feasible and effective [13,22]. UNHCR and other agencies should consider supporting refugee health staff and community members in establishing community-based organisations to provide curative and preventive sexual and reproductive health services within the refugee population.

## Competing interests

The authors declare that they have no competing interests.

## Authors' contributions

All authors reviewed and approved of the manuscript. In addition, the specific roles were as follows. MIC analysed the data and drafted the manuscript AvR conceived the study, contributed to its design and to the interpretation of the data. YS was involved in the conception, design and acquisition of data for the study. FvR contributed to the design of the study, and the interpretation of the data. AE contributed to analysis and interpretation of the data NH contributed to analysis, interpretation of data, and critical revision of the manuscript MB designed the study, contributed to the acquisition, analysis and interpretation of data, and critically revised the manuscript.

## Supplementary Material

Additional file 1 **Table 2 - Respondent's assessment of STI prevention methods (of those who had some knowledge of STIs).** The file "RHG Oct 08 table 2.doc" contains Table 2 which is in landscape format. Click here for file

Additional file 2 **Table 3 - Outcome indicators by source of STI information.** The file "RHG Oct 08 table 3.doc" contains Table 3 which is in landscape format. Click here for file
