# Supplementary material for: Reproductive health for refugees by refugees in Guinea II: sexually transmitted infections
Source: Confl Health. 2008 Oct 23;2:14. doi: 10.1186/1752-1505-2-14 (PMC2582230; doi:10.1186/1752-1505-2-14)
Supplement: Additional file 1 — Table 2 - Respondent's assessment of STI prevention methods (of those who had some knowledge of STIs). The file "RHG Oct 08 table 2.doc" contains Table 2 which is in landscape format. [file 1752-1505-2-14-S1.doc]

**Table 2 - Respondent’s assessment of STI prevention methods (of those who had some knowledge of STIs)**

|  | **% of men (N = 399)** | | |  | **% of women (N = 410)** | | | p-value, men vs women  (Chi^2^ test) |
| --- | --- | --- | --- | --- | --- | --- | --- | --- |
| **Methods posed to respondents** | **Agreed** | **Dis-agreed** | **Did not know** |  | **Agreed** | **Dis-agreed** | **Did not know** |  |
|  |  |  |  |  |  |  |  |  |
| ***Accepted methods of protection against STIs*** |  |  |  |  |  |  |  |  |
|  |  |  |  |  |  |  |  |  |
| - staying with one faithful partner | 91.5% | 5.3% | 3.3% |  | 93.9% | 2.2% | 3.9% | 0.064 |
|  |  |  |  |  |  |  |  |  |
| - using condoms during sexual intercourse | 91.5% | 2.0% | 6.5% |  | 91.2% | 2.4% | 6.3% | 0.913 |
|  |  |  |  |  |  |  |  |  |
| ***Inappropriate methods of protection against STIs*** |  |  |  |  |  |  |  |  |
|  |  |  |  |  |  |  |  |  |
| - swallowing a tablet before sexual intercourse | 42.1% | 38.3% | 19.5% |  | 32.4% | 51.7% | 15.9% | 0.001 |
|  |  |  |  |  |  |  |  |  |
| - avoiding public toilets | 42.9% | 44.4% | 12.8% |  | 44.4% | 41.7% | 13.9% | 0.730 |
|  |  |  |  |  |  |  |  |  |
| - for women to wash their genitals after sexual intercourse | 23.8% | 59.1% | 17.0% |  | 33.2% | 52.4% | 14.4% | 0.013 |
|  |  |  |  |  |  |  |  |  |
|  |  | | |  |  | | | p-value, men vs women  (Chi^2^ test) |
| **Summary responses** | **% of men (N = 399)** | | |  | **% of women (N = 410)** | | |  |
|  |  | | |  |  | | |  |
| ***Agreed with the two accepted methods of protection against STIs*** | 85.7% | | |  | 88.8% | | | 0.191 |
|  |  |  |  |  |  |  |  |  |
| ***Did not agree with any of the inappropriate methods of protection against STIs*** | 38.3% | | |  | 41.5% | | | 0.365 |
|  |  |  |  |  |  |  |  |  |
|  | | | | | | | |  |

## 
