# Supplementary material for: Reproductive health for refugees by refugees in Guinea II: sexually transmitted infections
Source: Confl Health. 2008 Oct 23;2:14. doi: 10.1186/1752-1505-2-14 (PMC2582230; doi:10.1186/1752-1505-2-14)
Supplement: Additional file 2 — Table 3 - Outcome indicators by source of STI information. The file "RHG Oct 08 table 3.doc" contains Table 3 which is in landscape format. [file 1752-1505-2-14-S2.doc]

## Table 3 - Outcome indicators by source of STI information

|  | **% of men** | | | | | **% of women** | | | | |
| --- | --- | --- | --- | --- | --- | --- | --- | --- | --- | --- |
|  | **by source of STI informationa** | | | | | **by source of STI informationa** | | | | |
|  | Non-healthcare sources | Health-care workers | RHG health facilitators | RHG drama groups | p-value | Non-healthcare sources | Health-care workers | RHG health facilitators | RHG drama groups | p-value |
| ***Respondents who had some knowledge of STIs*** | *(n = 98)* | *(n = 76)* | *(n = 193)* | *(n = 32)* |  | *(n = 45)* | *(n = 81)* | *(n = 256)* | *(n = 28)* |  |
|  |  |  |  |  |  |  |  |  |  |  |
| Knows key STI symptoms in both men and women | 8.2% | 14.5% | 31.6% | 43.8% | <0.001 | 17.8% | 14.8% | 30.1% | 32.1% | 0.022 |
|  |  |  |  |  |  |  |  |  |  |  |
| Agreed with the two accepted methods of protection against STIs | 77.6% | 85.5% | 91.7% | 75.0% | 0.003 | 68.9% | 91.4% | 91.4% | 89.3% | <0.001 |
|  |  |  |  |  |  |  |  |  |  |  |
| Did not agree with any of the inappropriate methods of protection against STIs | 31.6% | 22.4% | 45.1% | 56.3% | <0.001 | 24.4% | 30.9% | 47.3% | 46.4% | 0.005 |
|  |  |  |  |  |  |  |  |  |  |  |
|  |  |  |  |  |  |  |  |  |  |  |
| ***Respondents with genital discharge and/or ulceration in past 12 months*** | *(n = 26)* | *(n = 26)* | *(n = 47)* | *(n = 5)* |  | *(n = 13)* | *(n = 23)* | *(n = 85)* | *(n = 7)* |  |
|  |  |  |  |  |  |  |  |  |  |  |
| Stopped sex or used condoms, and notified partner/s§ | 69.2% | 80.8% | 83.0% | 60.0% | 0.408 | 38.5% | 47.8% | 50.6% | 42.9% | 0.858 |
|  |  |  |  |  |  |  |  |  |  |  |
| §: Includes individuals who: stopped sex and notified partners or used condoms and notified partners | | | | | | | | | | |
